# Potential Beneficial Effects of Extra Virgin Olive Oils Characterized by High Content in Minor Polar Compounds in Nephropathic Patients: A Pilot Study

**DOI:** 10.3390/molecules25204757

**Published:** 2020-10-16

**Authors:** Annalisa Romani, Roberta Bernini, Annalisa Noce, Silvia Urciuoli, Manuela Di Lauro, Anna Pietroboni Zaitseva, Giulia Marrone, Nicola Di Daniele

**Affiliations:** 1 PHYTOLAB (Pharmaceutical, Cosmetic, Food Supplement, Technology and Analysis), DiSIA, University of Florence, Via Ugo Schiff 6, Sesto Fiorentino, 50019 Florence, Italy; silvia.urciuoli@gmail.com; 2 Department of Agriculture and Forest Sciences (DAFNE), University of Tuscia, Via San Camillo de Lellis, 01100 Viterbo, Italy; 3 UOC of Internal Medicine-Center of Hypertension and Nephrology Unit, Department of Systems Medicine, University of Rome Tor Vergata, Via Montpellier 1, 00133 Rome, Italy; dilauromanuela@gmail.com (M.D.L.); annapietroboni@icloud.com (A.P.Z.); giul.marr@gmail.com (G.M.); didaniele@med.uniroma2.it (N.D.D.); 4 PhD School of Applied Medical, Surgical Sciences, University of Rome Tor Vergata, Via Montpellier 1, 00133 Rome, Italy

**Keywords:** extra virgin olive oil, minor polar compounds, phenolic compounds, hydroxytyrosol, in vivo studies, chronic kidney disease, nephropathic patients

## Abstract

Extra virgin olive oil (EVOO) is a lipid food, which constitutes a pillar of the Mediterranean diet. A high number of scientific data have demonstrated that it exerts a variety of beneficial effects on human health due to its peculiar chemical composition including fatty acids (98–99%) and other active compounds even if found in a very low percentage (1–2%). Among them, minor polar compounds (MCPs), represented mainly by phenolic compounds, are relevant for their healthy properties, as stated by the European Food Safety Authority’s (EFSA) claims. In this paper, we described the results obtained from a pilot in vivo study, focused for the first time on the evaluation of the possible beneficial effects of two EVOOs on chronic kidney disease (CKD) patients after the consumption of 40 mL *per* day for 9 weeks. The selected EVOOs, traced in the production chain, and characterized by High-Performance Liquid Chromatography (HPLC-DAD-MS) analysis, resulted rich in MCPs and satisfied the EFSA’s claim for their content of hydroxytyrosol and derivatives. The results obtained by this in vivo study appear to highlight the potential beneficial role in CKD patients of these EVOOs and are promising for future studies.

## 1. Introduction

Extra virgin olive oil (EVOO) is a food with unique organoleptic and pharmacological properties obtained by physical pressing of the drupes of *Olea europaea* L., a tree whose origin dates to the Paleolithic age. EVOO is a pillar of the Mediterranean diet, a dietary regimen traditional of the Mediterranean area responsible for the high longevity of the inhabitants and a low incidence of age-related diseases [1,2]. Extensive scientific research has demonstrated that daily consumption of EVOO is associated with numerous health benefits and a low-risk onset of chronic-degenerative non-communicable diseases (CDNCDs) [3]. Significant effects on the prevention of cancer, diabetes mellitus, chronic kidney disease (CKD), metabolic syndrome, obesity, arterial hypertension, neurological and cardiovascular diseases were reported [4,5,6,7]

These multiple beneficial effects are related to the presence of many active compounds found in EVOO responsible for its aroma, color, bitterness, and pungency. The qualitative and quantitative profile of these compounds depends on the place of cultivation, pedoclimatic conditions, variety and maturation stage of olives, and management practices to produce EVOO [8]. 

**Figure 1:**
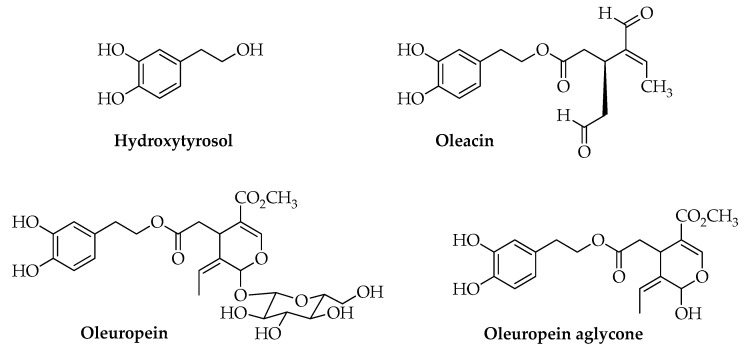
Chemical structures of hydroxytyrosol and derivatives found in EVOO.

Being a lipid matrix, the main active ingredients of EVOO are triacylglycerols (98–99%), mainly represented by oleic acid (55–83%), palmitic acid (7.5–20%), linoleic acid (3.5–21%), and other fatty acids (0.5–5%). Hydrocarbons, tocopherols, terpenes, sterols, chlorophylls, and minor polar compounds (MCPs) constitute the remaining percentage (1–2%) [9]. MCPs include phenolic compounds such as tyrosol, hydroxytyrosol, and its derivatives such as oleacin, oleuropein aglycone (Figure 1), lignans such as pinoresinol and acetoxypinoresinol, and non-phenolic compounds such as elenolic acid and its derivatives. 

Olive oil phenols have powerful antioxidant, anticancer, and anti-inflammatory activity [3,10,11,12,13,14,15,16,17,18]. The presence and content of these compounds are the basis of a health claim approved by the European Food Safety Authority (EFSA) in 2012 [19], which states that “olive oil polyphenols contribute to the protection of blood lipids from oxidative stress”. However, the same claim specifies that a daily intake of 20 g of an olive oil “containing at least 5 mg of hydroxytyrosol and its derivatives (e.g., oleuropein complex and tyrosol)” is necessary. This means that the beneficial effect on the oxidative stress of an EVOO was observed with the daily consumption of a product exhibiting at least 250 mg/kg of hydroxytyrosol and its derivatives [20].

Several studies have shown that MPCs present in EVOO display numerous beneficial actions on the human body; that they can reduce low-density lipoprotein- cholesterol (LDL-C) levels, oxidative damage to DNA, and inflammatory status [21,22,23]. Alterations of these parameters are frequent in the presence of CDNCDs. 

Although EVOO is a highly studied food, to date there are no human-based data that highlight its healthy action against CDNCDs by a randomized clinical trial. 

In this frame, this pilot study aimed to evaluate the possible beneficial effects of two EVOOs produced from organic cultivation on CKD patients after the assumption of 40 mL *per* day for 9 weeks. The EVOOs were previously characterized for their qualitative and quantitative content in MCPs by High-Performance Liquid Chromatography- with Diode Array Detector- Mass Spectrometry (HPLC-DAD-MS). Both EVOOs resulted rich in MCPs and satisfied the EFSA’s claim for their content of hydroxytyrosol and derivatives. To the best of our knowledge, this is the first in vivo study on CKD patients.

## 2. Results and Discussion

### 2.1. Chemical Characterization of EVOOs

The study was first focused on the chemical characterization of seven EVOOs derived from organic cultivation from Frantoio, Leccino, Moraiolo (Tuscany, Italy), and Intosso (Abruzzo, Italy) olive varieties. As summarized in Table 1, EVOOs named Frantoio (FR), Leccino (LE), Moraiolo (MO), Moraiolo (MOS), and Intosso (IN) were monocultivar, whereas Leccino (33.3%), Moraiolo (33.3%) and Frantonio (33.3%) was a Tuscan blend (TB) and Leccino (95%), Intosso (5%) was a Tuscan and Abruzzo blend (TAB). In particular, TB was a blend of three cultivars in equal parts (*v/v*): Frantoio, Leccino, and Moraiolo, in accordance to the Chianti (Tuscany) disciplinary; TAB was a blend of Leccino (95%, *v/v*) and Intosso (5%, *v/v*). 

All olive trees were grown organically; those from which MOS was obtained were also under copper-free conditions. Olives were crushed in the olive oil mills of MORI-TEM (Florence, Italy) using innovative equipment to obtain good quality and low oxidation impact products (see Materials and Methods, Section 3.2).

Acidity, peroxides, and polyphenols were determined for all EVOOs using the OxiTester Analysis System—a fast, simple, and reliable analyzer based on a photometric technology. The content of polyphenols was related to the antioxidant capacity of the sample. A study carried out in 2008 has validated the data of the antioxidant capacity obtained by the OxiTester analyzer of a wide range of EVOOs samples with those of the official method—the Rancimat method [24].

As reported in Table 2, the acidity, expressed as a percentage of oleic acid, ranged from 0.15 for LE, MOS, and IN to 0.25 for FR; the peroxides, reported in meqO_2_/Kg, varied from 4.49 for IN to 8.80 for TB; the polyphenols, expressed as mg tyrosol/Kg, reached for all samples significant values varying from 342 for TB to 890 for TAB. Among monocultivar EVOOs, LE was characterized by the highest value (791 mg tyrosol/Kg) while IN by the lowest value (400 mg tyrosol/Kg).

After the preliminary analysis with the OxiTester system, all EVOOs were analyzed by HPLC-DAD-MS to identify and quantify the single MCPs found in each sample. As reported in Table 3, the highest value of MCPs was found for TAB and LE, respectively with 1145.98 and 891.70 mg/L, followed by MOS (706.83 mg/L), MO (686.96 mg/L), FR (654.90 mg/L), TB (496.80 mg/L), and IN (417.96 mg/L). A similar trend was observed for the total phenolic compounds; noteworthy is the high content of lignans in all samples and, in particular, TAB and MO with 208.17 and 205.02 mg/L, respectively. FR, LE, MO, MOS, TB, and TAB satisfy the above reported EFSA’s health claim with the highest value of hydroxytyrosol and derivatives content for TAB (986.60 mg/L), LE (799.36 mg/L), and MOS (351.25 mg/L).

Based on the chemical characterization of all samples, the selected EVOOs for the in vivo study in CKD patients were MOS and TB, characterized by a different MCPs content, respectively 706.83 and 496.80 mg/L. These samples showed values of total phenolic compounds of 480.73 and 358.87 mg/L and a content of hydroxytyrosol and derivatives of 351.25 and 296.73 mg/L, respectively (Table 3). MOS was selected despite being a monocultivar EVOO produced from organic cultivation under copper-free conditions, while TB is a blend of three different cultivars (Leccino, Moraiolo, and Frantoio). IN was not chosen for the lowest value of total phenolic compounds (310.75 mg/L) and hydroxytyrosol and derivatives content (216.08 mg/L), which does not respect the health claim [19]. However, TAB was not chosen for the lack of literature about possible in vivo pro-oxidative effects of EVOOs characterized by a high polyphenol and hydroxytyrosol content. 

For CKD patients, MOS and TB are included in their daily diet as the only plant lipid source.

### 2.2. Human Study 

The main epidemiological characteristics of the two groups of patients are summarized in Table 4. Patients assumed two EVOOs at different MCP content: 14 patients (age mean 70.8 ± 12.4 years) took MOS and 13 patients (age mean 65.9 ± 11.4 years) took TB.

Primary causes of CKD were chronic glomerulonephritis (7.4%), nephroangiosclerosis (37.2%), diabetic nephropathy (11%), chronic pyelonephritis (7.4%), and other causes (37%).

The results of the laboratory parameters are reported in Table 5. We observed at T1, compared to T0, a significant increase of estimated glomerular filtration rate (e-GFR) in the group of patients that consumed MOS (35.4 ± 16.32 mL/min vs. 38.1 ± 16.69 mL/min; *p* = 0.04). 

In the same group, we observed a significant decrease of uric acid (6.36 ± 1.9 mg/dL vs. 5.0 ± 1.2 mg/dL, *p* = 0.049) and an improvement of lipid profile with a significant decrease of triglycerides (165.75 ± 83.18 mg/dL vs. 145.08 ± 70.9 mg/dL, *p* = 0.016). 

In both groups, we observed a significant increase of serum albumin (4.17 ± 0.26 g/dL vs. 4.31 ± 0.27 g/dL, *p* = 0.021; 4.12 ± 0.25 g/dL vs. 4.39 ± 0.38 g/dL; *p* = 0.032). 

In both groups, we have not observed any statistically significant differences for the atherogenic and inflammatory indices.

**Figure 2:**
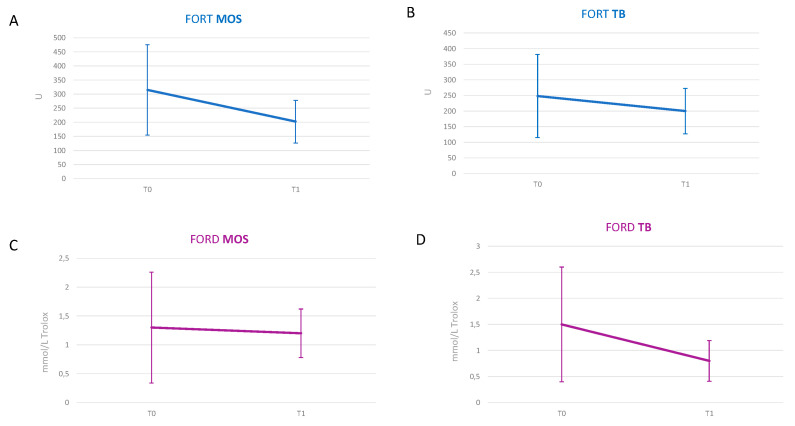
Graphic representation of oxidative parameters in the two time-points of the stud: (**A**) trend of FORT values in patients treated with MOS EVOO, (**B**) trend of FORT values in patients treated with TB EVOO, (**C**) trend of FORD values in patients treated with MOS EVOO, (**D**) trend of FORD values in patients treated with TB EVOO. *Abbreviations: EVOO, extra virgin olive oil; FORT, Free Oxygen Radical Test; FORD, Free Oxygen Radical Defense; MOS, Moraiolo (100%); TB, Leccino (33.3%), Moraiolo (33.3%), Frantoio (33.3%).*

At the end of the study, we observed a trend of reduction of the Free Oxygen Radical Test (FORT) and Free Oxygen Radical Defense (FORD), although not significant, in both groups, as reported in Figure 2.

Statistical analysis shows no significant changes in eating habits, monitored through the Prevención con Dieta Mediterránea (PREDIMED) questionnaire, administered at two times of the study (T0 and T1), and in physical activity, monitored through the International Physical Activity Questionnaire (IPAQ), administered at the same time-points (Table 6 and Table 7). Therefore, there is no statistically significant difference between the scoring of both questionnaires administered in both groups. This makes the results obtained from the study even more reliable as they are not influenced by modifiable factors such as dietary habits and degree of physical activity.

The preliminary data obtained from the present study seem to highlight the potential beneficial role of MOS, the EVOO characterized by a high value of MPCs in CKD patients. In fact, the intake of 40 mL *per* day of MOS would seem to increase the e-GFR after 9 weeks. This data could be supported by the observed reduction trend of oxidative stress (OS); in fact, the latter plays a key role in the progression of the CKD because OS is related to CKD complications like arterial hypertension, low-grade chronic inflammation, and anemia [25,26]. The OS reduction observed confirms previous studies [27,28,29,30] such as the CMPs of EVOO play a key role in the mechanisms that favor the induction of antioxidant activity [31,32,33].

The GFR improvement could also be related to the statistically significant reduction in uric acid levels that we observed in patients treated with 40 mL *per* day of MOS, after 9 weeks of assumption. In this regard, many studies have shown that the use of urate-lowering therapy is related to the slowing of the progression of CKD [34,35,36,37].

Previous in vivo studies demonstrated that EVOO can improve the lipid profile by the enhancement of high-density lipoprotein-cholesterol (HDL-C) and the reduction of LDL-C and triglyceride concentration [38,39,40]. This reduction seems to be related to the concentration of EVOO MPCs, as reported by several authors [38,41,42].

Our data confirm a significant reduction of triglyceride levels due to the consumption of 40 mL *per* day of EVOO (MOS) in CKD patients.

## 3. Materials and Methods 

### 3.1. Reagents

All chemicals used were of analytical grade. Solvents and reagents were purchased from Sigma Aldrich (Milan, Italy). Tyrosol and oleuropein were furnished by Extrasynthèse, (France). Hydroxytyrosol was available by synthesis [43]. Water was purified by a Milli-Q Plus system from Millipore (Milford, MA, USA).

### 3.2. EVOOs Production 

Five EVOOs were monocultivar (FR; LE; MO, and MOS; IN) and two were blends (TB and TAB). All EVOOS were organic, MOS was grown also copper-free. The EVOOs were produced at the end of October 2018 from a single farmer of Vinci (Florence, Italy) using the olive oil mill MORI-TEM (Florence, Italy) by CULTIVAR equipment. The crusher was separated and provided with a mechanical apparatus for the turns speed regulation. Malaxation was made inside closed, vertical malaxers, with loading from below, air intake, and presence of a modified atmosphere with helium and nitrogen (50/50 *v/v*) and low concentration of oxygen (less than 10%). The temperature was checked is in all process phases: the first step of malaxing was carried out at 3000 rpm at 30 °C for 20 min; the second step at 5000 rpm at 32 °C for 5 min. Pressing was carried out in the presence of 65% nitrogen. After the preparation, all EVOOs were bottled, corked, and kept away from sources of heat and light.

### 3.3. Determination of Acidity, Peroxides, and Total Polyphenols 

The acidity, peroxides, and polyphenols of all samples were determined using the CDR OxiTester Analysis System (CDR srl Florence, Italy) and specific reagent kits. The acidity was expressed as a percentage of oleic acid. By the reaction of the fatty acids present in the sample with a chromogen at pH < 7.0, the color was developed; the optical density, measured at 630 nm, was proportional to the concentration of the fatty acids. The peroxide value was expressed as meqO_2_/Kg. The quantification was based on the ability of peroxides to oxidize Fe^2+^ to Fe^+3^, responsible for the formation of a red complex. The intensity, measured at 505 nm, was directly proportional to the concentration of the peroxide value in the sample. The total polyphenols, expressed in mg tyrosol/Kg, were quantified using a chromogen dissolved in an alcoholic solution. Under these conditions, polyphenols were oxidized causing a decrease in the color of the solution. The concentration of polyphenols in the sample was determined by measuring the optical density of the solution at 505 nm [44] using a calibration curve directly updated on the instrumentation, correlated to the Folin–Ciocalteau method applied to the same standard (tyrosol).

### 3.4. HPLC-DAD-MS Analyses of EVOOs

All EVOOs were first treated with hexane to remove the lipid fraction. Then, 25 mL of each sample was extracted with 75 mL of EtOH/H_2_O = 70:30 (*v/v*) using water that was acidified with formic acid (pH = 3.2). The hydroalcoholic solvent was evaporated under reduced pressure to dryness using a Rotavapor (BUCHI, Germany); then, the residue was dissolved with 4 mL of EtOH/H_2_O (pH 3.2) = 70:30 (*v/v*) and analyzed using an HP-1260 Infinity II liquid chromatograph equipped with a DAD detector and an Infinity Lab LC/MSD quadrupole mass spectrometer with an API-electrospray interface (Agilent Technologies, Palo Alto, CA, USA). The HPLC-DAD-MS method used was developed by our research team in 2014 [9]. The single compounds were quantified by a four-point regression curve using tyrosol and oleuropein as standard. Calibration curves with an r^2^ ≥ 0.9998 were used. Tyrosol and lignans concentrations were quantified at 280 nm using tyrosol as the reference compound: the secoiridoids at 280 nm, elenolic acid, and elenolic acid derivatives at 240 nm using oleuropein as the reference compound. In all cases, the concentrations of derivatives were calculated after applying the appropriate correction depending on the molecular weight: knowing the molecular weight of each compound (MWx), their concentration was obtained by applying a multiplication factor of MWx/MWy, where MWy is the molecular weight of the specific reference compound.

### 3.5. Patients and Methods 

A group of 27 patients affected by CKD, stage I–IV according to the National Kidney Foundation Kidney Disease Outcomes Quality Initiative guidelines [45], in conservative therapy, were enrolled at the UOC of Internal Medicine, Center for Hypertension and Nephrology Unit of the University Hospital Policlinico Tor Vergata (PTV), Rome. 

The study protocol was declared compliant with the Helsinki declaration by the PTV Independent Ethics Committee. All enrolled subjects signed an informed consent form at the enrollment. At enrollment, a complete medical history was collected for each patient. The inclusion criteria were an age between 18 and 80 years of either sex. The exclusion criteria were the presence of cancer in the active phase, human immunodeficiency virus (HIV), hepatitis B surface antigen (HbsAg^+^), human hepatitis C virus (HCV^+^), inflammatory, and/or infectious pathologies in the acute phase; BMI < 18 kg/m^2^; pregnancy and breastfeeding. All patients followed an Italian standard Mediterranean diet with a controlled protein intake according to the CKD stage [46,47].

All enrolled patients were instructed to consume 40 mL *per* day of EVOO, for a total time of 9 weeks. During this period, the pharmacological therapy of all patients enrolled in the study, did not change. Evaluation of laboratory parameters was conducted at two different times of the study, at baseline (T0) and after 9 weeks (T1) of EVOO assumption.

### 3.6. Questionnaires

In order to exclude possible biases induced by eating habits changing and the degree of physical activity, two questionnaires were administered to all patients at the two time-points of the study (T0 and T1): PREDIMED, which assessed the degree of adherence to the Mediterranean diet [48] and the IPAQ, which assessed the degree of weekly physical activity [49].

### 3.7. Laboratory Parameters

At T0 and T1, the evaluation of blood and urinary parameters (such as azotemia, creatinine, uricemia, electrolytes, chemical–physical urinary examination, and albuminuria on morning urine) were performed. The evaluation of the cardiovascular risk indices was assessed as follows: lipid profile (Total cholesterol-TC, HDL-C, LDL-C, and triglycerides) and atherogenic indices (TC/ HDL-C, LDL-C/ HDL-C, Log Triglycerides / HDL-C). The assessment of OS and antioxidant defense using the FORT and FORD performed with the use of the CR4000. The latter is a rapid analytical technique, through a capillary sampling. In particular, the FORT method measures the levels of circulating oxygen free radicals [50], while the FORD is an indirect measure of antioxidant capacity, evaluated by the concentration of ascorbic acid, albumin, and glutathione [51]. 

FORD and FORT simultaneous monitoring allow for evaluating the effectiveness of the individual’s antioxidant defense mechanisms.

Inflammatory indices such as C-reactive protein (CRP), erythrocyte sedimentation rate (ESR), platelet-to-lymphocyte ratio, neutrophil-to-lymphocyte ratio, and lymphocyte-to-monocyte ratio were also monitored.

All parameters were monitored by Dimension Vista 1500 (Siemens Healthcare Diagnostics, Milano, Italy). The lipid profile was assessed by standard enzymatic colorimetric techniques (Roche Modular P800, Roche Diagnostics, Indianapolis, IN, USA). All other parameters were analyzed according to standard procedures in the Clinical Chemical Laboratories of University hospital PTV of Rome.

### 3.8. Statistical Analysis

All data were initially entered into an Excel spreadsheet (Microsoft, Redmond, Washington, United States) and the analysis was performed using the Windows social sciences statistical package, version 15.0 (SPSS, Chicago, Illinois, USA). The descriptive statistic consisted of the mean ± standard deviation (SD) for normally distributed variables (after confirmation with histograms and the Kolgomorov–Smirnov test), or as the median and range (min; max) For parameters not normally distributed. Occurrences, on the other hand, were assessed as percentages. Frequency comparisons between treatment groups were performed with chi-square tests. The comparison of homogeneity between normal variables between the treatments was carried out with a one-way ANOVA, while the comparisons between treatments over time (T0; T1) were conducted with *t*-test paired or between oils and time with ANOVA for repeated measurements. A *p*-value < 0.05 was considered statistically significant.

## 4. Conclusions

In this study, seven EVOOs derived from organic cultivation, named FR, LE, MO, MOS, IN, TB, and TAB, traced in the production chain, were chemically characterized. Acidity, peroxides, and polyphenols were determined using the CDR OxiTester Analysis System, a fast, simple, and reliable analyzer. Then, they were characterized by HPLC-MS-DAD to identify and quantify all MCPs found in each sample.

Based on the chemical characterization, two EVOOs with a different MCPs content were selected for the pilot in vivo study described in this paper: MOS and TB (with MCP of 706.83 and 496.80 mg/L, respectively).

The preliminary results obtained by the clinical study highlighted the potential beneficial effects of EVOO MCPs in CKD patients. In fact, the chronic consumption of 40 mL *per* day of EVOO with MCPs high content, seems to be useful in the clinical management of nephropathy.

Further studies conducted on a larger sample and with a longer follow-up period are needed to confirm the results obtained. Moreover, it would be interesting to observe the trend of the laboratory parameters after a washout period.

Currently, based on the preliminary results obtained by this research, a clinical study is in progress to evaluate the effect of the other EVOOs reported in Table 1 as TAB, characterized by a higher content of MPCs concerning MOS and TB. Specifically, this study aims to evaluate the potential antioxidant and anti-inflammatory effects of TAB, due to its high content in oleocanthal and its derivatives. The population of CKD patients was also selected because renal disease is known to be characterized by an inflammatory state inversely related to the renal function [52].

## Figures and Tables

**Table 1:** EVOOs samples.

Sample	Cultivar (%, *v/v*)
FR	Frantoio (100)
LE	Leccino (100)
MO	Moraiolo (100)
MOS	Moraiolo (100)
IN	Intosso (100)
TB	Leccino (33.3), Moraiolo (33.3), Frantoio (33.3)
TAB	Leccino (95), Intosso (5)

**Table 2:** Acidity, peroxides, and polyphenols of EVOOs samples *.

	Acidity (% oleic acid)	Peroxides (meqO_2_/Kg)	Polyphenols (mg tyrosol/Kg)
FR	0.25	6.67	545
LE	0.15	5.21	791
MO	0.24	5.80	717
MOS	0.15	7.81	483
IN	0.15	4.49	400
TB	0.16	8.80	342
TAB	0.17	4.98	890

* All results are the average of three determinations and the standard error was <3% for acidity; <5% for peroxide value and <10% for polyphenols.

**Table 3:** High-Performance Liquid Chromatography (HPLC-DAD-MS) analysis of EVOOs samples.

Compound	FR	LE	MO	MOS	IN	TB	TAB
	mg/L *						
Hydroxytyrosol	0.80	nd	7.04	1.88	2.06	1.47	3.10
Tyrosol	0.98	nd	4.00	1.87	3.76	1.86	1.02
Elenolic acid	198.98	31.54	129.71	196.78	101.41	116.73	150.06
Elenolic acid derivatives	31.40	60.80	22.24	29.32	5.80	21.20	9.32
Oleacin	154.81	361.89	45.51	123.58	54.06	67.47	315.46
Oleocanthal	44.37	192.42	40.77	44.03	54.91	94.12	197.84
Oleuropein aglycone	84.14	67.70	143.12	143.46	64.75	83.42	164.58
Secoiridoids derivatives	40.33	17.11	89.55	36.43	36.54	48.39	96.43
Lignans	99.09	160.25	205.02	129.48	94.67	62.14	208.17
*Total MCP*	654.90	891.71	686.96	706.83	417.96	496.80	1145.98
*Total phenolic compounds*	424.52	799.37	535.01	480.73	310.75	358.87	986.60
*Total hydroxytyrosol and derivatives*	325.43	639.12	329.99	351.25	216.08	296.73	778.43

* All results are the average of three determinations and the standard error is <2.5%; nd = not detected.

**Table 4:** Description of study population and evaluation of the homogeneity of the study groups.

	MOS	TB	p (ANOVA Test)
**N**	14	13	
**Gender (male/female)**	4/10	4/9	ns
**Age (years)**	70.8 ± 12.4 ^a^	65.9 ± 11.4 ^a^	ns
**Height (m)**	1.65 ± 0.11 ^a^	1.66 ± 0.12 ^a^	ns
**Weight (kg)**	78.9 ± 13.9 ^a^	75.6 ± 15.6 ^a^	ns
**BMI (kg/m^2^)**	28.83 ± 3.86 ^a^	27.43 ± 5.81 ^a^	ns

^a^ Data expressed as mean ± standard deviation; ns = not significant.

**Table 5:** Laboratory parameters of Moraiolo (MOS) and TB groups.

		MOS			TB	
	T0	T1	T0 vs. T1	T0	T1	T0 vs. T1
**Creatinine (mg/dL)**	2.04 ± 0.68 ^a^	1.9 ± 0.68 ^a^	ns ^b^	2.22 ± 1.26 ^a^	2.17 ± 1.26 ^a^	ns ^b^
**e-GFR** **(mL/min/1.72 m^2^)**	35.4 ± 16.32 ^a^	38.1 ± 16.69 ^a^	0.04 ^b^	37 ± 20.84 ^a^	39.11 ± 22.95 ^a^	ns ^b^
**Albuminuria (mg/dL)**	9.2 ± 16.2 ^a^	20.4 ± 37.8 ^a^	ns ^b^	25 ± 63.7 ^a^	6 ± 10.49 ^a^	ns ^b^
**Albumin (g/dL)**	4.17 ± 0.26 ^a^	4.31 ± 0.27 ^a^	0.021 ^b^	4.12 ± 0.25 ^a^	4.39 ± 0.38 ^a^	0.032 ^b^
**Azotemia (mg/dL)**	69.5 ± 24.2 ^a^	61.8 ± 20.6 ^a^	ns ^b^	61.09 ± 18.41 ^a^	63.9 ± 24.13 ^a^	ns ^b^
**Sodium (mEq/L)**	139.8 ± 3.6 ^a^	139.9 ± 2.0 ^a^	ns ^b^	141.64 ± 2.06 ^a^	140.09 ± 2.74 ^a^	ns ^b^
**Potassium (mEq/L)**	4.41 ± 0.56 ^a^	4.35 ± 0.61 ^a^	ns ^b^	4.44 ± 0.43 ^a^	4.31 ± 0.4 ^a^	ns ^b^
**Calcium (mg/dL)**	9.92 ± 0.43 ^a^	9.72 ± 0.38 ^a^	ns ^b^	9.85 ± 0.46 ^a^	9.7 ± 0.47 ^a^	ns ^b^
**Phosphorus (mg/dL)**	3.39 ± 0.49 ^a^	3.27 ± 0.54 ^a^	ns ^b^	3.61 ± 0.61 ^a^	3.53 ± 0.67 ^a^	ns ^b^
**TC (mg/dL)**	176.58 ± 44.17 ^a^	171.9 ± 38.0 ^a^	ns ^b^	173.64 ± 59.61 ^a^	180.73 ± 52.4 ^a^	ns ^b^
**HDL-C (mg/dL)**	40.08 ± 9.59 ^a^	56.4 ± 37.1 ^a^	ns ^b^	45 ± 11.79 ^a^	48.45 ± 12.88 ^a^	ns ^b^
**LDL-C (mg/dL)**	102.25 ± 31.85 ^a^	94.5 ± 34.09 ^a^	ns ^b^	109.9 ± 51.9 ^a^	100.3 ± 41.6 ^a^	ns ^b^
**Triglycerides (mg/dL)**	165.75 ± 83.18 ^a^	145.08 ± 70.9 ^a^	0.016 ^b^	114.5 ± 69.6 ^a^	117.8 ± 59.8 ^a^	ns ^b^
**Sideremia (μg/dL)**	89.08 ± 36.47 ^a^	128 ± 170.0 ^a^	ns ^b^	76.27 ± 23.48 ^a^	71.55 ± 20.71 ^a^	ns ^b^
**Glycaemia (mg/dL)**	96.6 ± 25 ^a^	101.0 ± 36.2 ^a^	ns ^b^	94.09 ± 21.54 ^a^	90.36 ± 13.8 ^a^	ns ^b^
**Uric acid (mg/dL)**	6.36 ± 1.9 ^a^	5.0 ± 1.2 ^a^	0.049 ^b^	6.23 ± 1.63 ^a^	6.1 ± 0.86 ^a^	ns ^b^
**PTH (pg/mL)**	83.7 ± 42.7 ^a^	82.56 ± 45.9 ^a^	ns ^b^	81.08 ± 60.65 ^a^	72.38 ± 29.1 ^a^	ns ^b^
**CRP (mg/L)**	4.48 ± 1.2 ^a^	3.61 + 0.8 ^a^	ns ^b^	2.92 + 0.98 ^a^	2.77 + 1.3 ^a^	ns ^b^
**ESR (mm/h)**	34.91 ± 23.65 ^a^	32.36 ± 27.75 ^a^	ns ^b^	40.28 ± 30.1 ^a^	31 ± 28.65 ^a^	ns ^b^

^a^ Data expressed as mean ± standard deviation; ^b^ Applied test: *t*-test for paired data. Values of *p* ≤ 0.05 are considered statistically significant. *Abbreviations: e-GFR, estimated glomerular filtration rate; TC, total cholesterol; HDL-C, high-density lipoprotein cholesterol; LDL-C, low-density lipoprotein cholesterol; CRP, C-reactive protein; ESR, erythrocyte sedimentation rate; PTH, parathyroid hormone;* *ns, not significant.*

**Table 6:** Prevención con Dieta Mediterránea (PREDIMED) questionnaire.

	MOS			TB		
	T0	T1	p (McNemar’s Test)	T0	T1	p (McNemar’s Test)
**Minimal adherence (%)**	0 (0)	2 (15.3)	ns	1 (7.7)	0 (0)	ns
**Average adherence (%)**	9 (64.3)	7 (54)	ns	8 (61.6)	9 (64.3)	ns
**Maximal adherence (%)**	5 (35.7)	4 (30.7)	ns	4 (30.7)	5 (35.7)	ns

ns = not significant.

**Table 7:** International Physical Activity Questionnaire (IPAQ).

	MOS			TB		
	T0	T1	p (McNemar’s Test)	T0	T1	p (McNemar’s Test)
**Inactive (%)**	5 (35.7)	9 (69.4)	ns	8 (61.5)	5 (35.7)	ns
**Sufficiently active (%)**	6 (35.7)	2 (15.3)	ns	3 (23.2)	6 (35.7)	ns
**Very active (%)**	3 (21.5)	2 (15.3)	ns	2 (15.3)	3 (21.5)	ns

ns = not significant.
